# How often do healthy people cough?

**DOI:** 10.1186/s12931-023-02585-1

**Published:** 2023-11-11

**Authors:** Kimberley J. Holt, Jaclyn A. Smith

**Affiliations:** 1 Division of Infection, Immunity and Respiratory Medicine, University of Manchester and Manchester Academic Health Science Centre, Manchester, UK; 2 Manchester University NHS Foundation Trust, Manchester, UK

**Keywords:** Objective cough frequency, Cough monitoring, Cough quantification, Healthy controls.

## Abstract

Objective cough frequency has been reported in several respiratory conditions but the amount that healthy individuals cough daily is unclear. Seventy-nine healthy volunteers (38 males, median [IQR] age 41y [IQR 30–53]) completed 24-hour ambulatory cough monitoring (VitaloJAK™). The audio recording was filtered using a custom written algorithm to remove non-cough sounds and then all individual explosive cough sounds in the filtered file were tagged electronically by trained cough counters. Most coughing occurred during the day and cough numbers over 24 h were generally low (geometric mean of 4.6 coughs) but there was large variability; ranging from 0 to 136 coughs overall. Cough frequency was independent of participant characteristics apart from sex with males coughing significantly, 4–5 fold, more than females during the day and over 24 h (median [IQR] 16.1 [3.8–33.4] vs. 4.1 [1.0–15.0] total coughs; p = 0.015). This is the first report to describe cough frequency in a balanced group of healthy adults using an accurate cough monitoring system. The data reveal a further example of sexual dimorphism in cough, which warrants additional investigation.

## Correspondence

Developments in cough quantification systems have significantly improved the way new treatments for cough are evaluated and cough mechanisms are studied. Objective 24-hour cough frequency has been measured in patients with various respiratory conditions [1–4] however it is unclear how much healthy people cough daily. It would be useful to understand normal cough frequency for several reasons. There may be clinical value in identifying thresholds at which cough frequency is more likely to be excessive and therefore represents pathology. In addition, there has recently been significant development in novel medications for the treatment of chronic cough and such a threshold may be helpful in determining patient inclusion criteria for clinical trials [5]. Furthermore, in respiratory disease outbreaks such as the COVID-19 pandemic, the frequency of coughing in health suggests the potential for transmission of virus by asymptomatic or pre-symptomatic individuals.

Only a few previous studies have reported spontaneous cough frequency in healthy volunteers but they were carried out in small sample sizes, using cough monitoring devices not fully validated, and one study was in children [1, 6, 7]. Therefore, the aim of this analysis was to accurately report cough frequency in a group of healthy people and evaluate the influence of participant characteristics such as age and sex. The data for this report were collected as part of a study reported previously [8] investigating cough responses to inhaled irritants (REC ref; 13/NW/0400). Cough monitoring was carried out at baseline, prior to any cough challenge testing. All participants provided written informed consent.

Healthy volunteers were recruited from staff, students and databases of volunteers who had previously consented to be approached for future research. Inclusion criteria comprised normal spirometry (FEV1 and FVC ≥ 80% predicted), no recent respiratory tract infection (< 4 weeks) and no significant current or past medical history that in the opinion of the investigators would affect the study (including respiratory disease, chronic cough, chronic pain, irritable bowel syndrome, psychiatric illness, chronic headaches, diabetes, cardiac or cerebrovascular disease). Current smokers, ex-smokers with > 20 pack year history and/or < 6 months abstinence and those taking cough-modulating medications (including angiotensin-converting enzyme inhibitors, opiates and centrally acting antidepressants) were excluded.

Participants were fitted with an ambulatory cough monitor (VitaloJAK™, Vitalograph, UK) for 24 h. At the end of the recording, the audio file was filtered by a custom-written algorithm to remove non-cough sounds and silence [9] and then analysed by trained cough counters who manually placed electronic tags on each individual explosive cough sound. The algorithm retains a median 100% of coughs (IQR 99.5–100) [9] and agreement for cough counting between trained cough analysts is excellent [10]. The number of cough tags was totalled and the times that participants retired to bed and woke up were determined by listening to sections of the 24 h audio recording in order to calculate daytime and night-time cough frequency.

Data were summarised using means (standard deviation), medians (interquartile range), geometric means (95% confidence intervals) and ranges. The influence of sex was analysed using Mann-Whitney U tests. Cough frequencies were correlated (Spearman’s) with participant characteristics (age, BMI, smoking history, lung function). The upper limit of normal for cough frequency was estimated by calculating 95th percentiles.

**Fig. 1:**
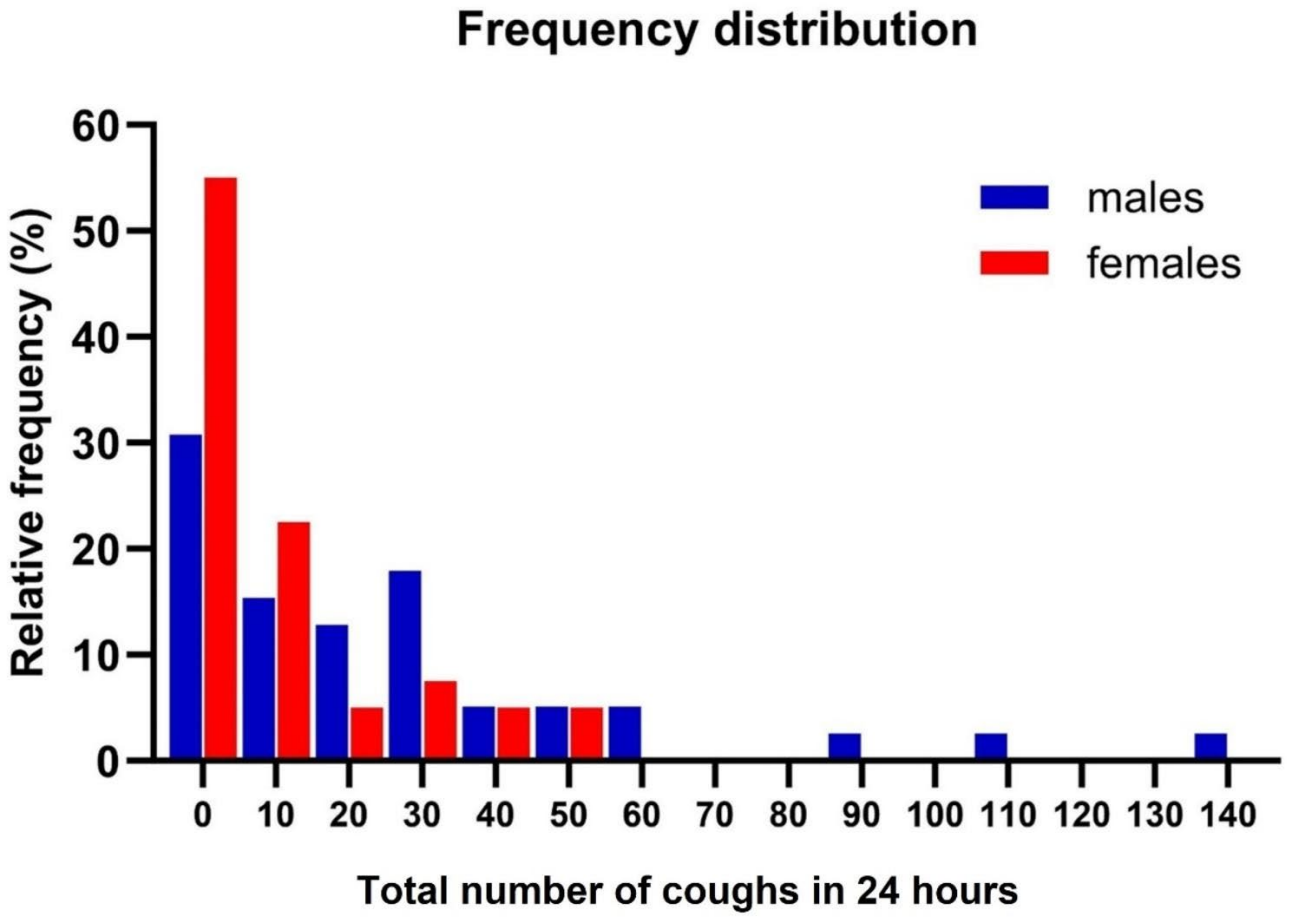
Frequency distribution of total numbers of coughs in 24 h comparing males with females. Relative frequency was calculated as a proportion of the total number of participants for each gender (n = 38 females and n = 41 males)

Seventy-nine healthy participants (38 males) were enrolled; median (IQR) age 41y (IQR 30–53, range 20–74); BMI 25.4 (22.4–28.6). Lung function was normal; median(IQR) FEV1 and FVC were 104 (96–115) and 107 (100–121) % predicted and smoking history was minimal; median(IQR) pack year history was 0.0 (0.0–0.0) with 14 volunteers being former smokers. Most participants had low cough numbers over 24 h; a median total count of 7 coughs; 0.3 coughs/hour (c/h) (Table 1). Three quarters of subjects coughed less than 30 times and eight people (~ 10%) did not cough at all in 24 h. However, there was considerable variability in the numbers of coughs recorded (Fig. 1), particularly in males who exhibited 4-5-fold more coughs than females during the day and over 24 h (median [IQR] 16.1 [3.8–33.4] vs. 4.1 [1.0–15.0] total coughs; p = 0.015). Most coughing occurred during the day; night-time cough was scarce and did not differ between sexes. There were no significant correlations between cough frequency and any of the characteristics recorded (age/BMI/smoking history/FEV1%/FVC%), except that BMI weakly correlated with the coughing at night (r = 0.3, p = 0.007).

**Table 1:** Total (24 h), daytime and night-time cough frequencies for males (n = 38), females (n = 41) and all 79 participants combined; displayed as mean (standard deviation; SD), geometric mean (95% confidence interval; CI), median (interquartile range; IQR) and range (minimum to maximum values). P values indicate differences between sexes compared using Mann-Whitney U test

		Mean (SD)	Geometric Mean (95% CI)	Median (IQR)	Range (Min-Max)	95th Percentile
***Total (24 h) coughs***	**All**	18.2 (25.0)	4.6 (2.7-8.0)	7.0 (1.9–29.0)	0.0-136	63.1
	**Male**	25.5 (31.3)	7.9 (3.7–17.1)	16.1 (3.8–33.4)	0.0-136	111.2
	**Female**	11.3 (14.5)	2.8 (1.3-6.0)	4.1 (1.0–15.0)	0.0–51	44.8
	***p value***			***0.015***		
***Daytime coughs***	**All**	16.7 (23.6)	3.4 (1.8–6.3)	7.0 (2.0–7.0)	0.0-136	63
	**Male**	23.8 (30.0)	7.2 (3.3–15.6)	15.0 (3.8–30.8)	0.0-136	98
	**Female**	10.1 (12.8)	1.7 (0.7–4.2)	3.0 (1.0–14.0)	0.0–42	38.9
	***p value***			***0.014***		
***Night-time coughs***	**All**	1.4 (2.9)	0.1 (0.0-0.1)	0.0 (0.0–1.0)	0.0–14	8
	**Male**	1.7 (3.2)	0.1 (0.0-0.2)	0.0 (0.0-2.3)	0.0–14	10.2
	**Female**	1.2 (2.7)	0.1 (0.0-0.1)	0.0 (0.0–1.0)	0.0–12	7.9
	***p value***			*0.676*		

Based on the 95th percentile, the upper limit of normal for total cough frequency was 63 coughs over 24 h (2.6 c/h). Due to the differences observed between sexes, the upper limits calculated separately for males and females were 111 (4.6 c/h) and 45 (1.9c/h) coughs respectively.

This is the first report to present 24-hour objective cough frequency in a large group of adult healthy controls. Cough frequency was accurately quantified using an FDA approved and CE marked, semi-automated cough monitoring system, validated in several disease groups including healthy volunteers [9]. The amount of coughing was quite variable with some individuals not coughing at all whilst 136 coughs were recorded in one individual. In keeping with previous findings, most coughing occurred during waking hours, with very little at night. Cough frequency was independent of participant characteristics apart from sex.

Notably, males coughed significantly more than females during waking hours, and over 24 h. This contrasts with studies of cough responses to inhaled irritants such as capsaicin which have shown healthy females have a more sensitive and responsive cough reflex when evoked experimentally [11]. Indeed, specialist cough clinics are predominantly attended by females who also exhibit significantly higher cough frequencies than men with chronic cough [11]. It is impossible to explain this difference from our study, however perhaps sub-clinical or undiagnosed conditions were more prevalent in the males than females. The correlation between overnight cough and BMI might implicate conditions such as obstructive sleep apnoea and gastro-oesophageal reflux.

This study has some limitations. Participants were only recorded on a single occasion and so repeatability of cough frequency in healthy volunteers was not established. Also, it is possible that the awareness of having their cough monitored altered the amount of coughing observed.

To the best of our knowledge, only two previous studies have quantified spontaneous cough frequency in healthy adults. Hsu et al. studied 12, mostly male, healthy controls only reporting a range of 0–16 coughs per day [1]. As no further information was reported, it is difficult to compare this data to our findings. More recently, Yousaf and colleagues reported a geometric mean of 18.6 coughs per 24 h in 44 (27% female) healthy subjects [7]. In the latter study, females coughed more than males however, as acknowledged in the report, the sensitivity for cough detection was only 83.8% and the relatively large number of false positives likely inflated the cough counts. Our dataset is larger and more balanced for sex compared with previous studies and, together with precise cough monitoring provides the most accurate estimate of cough frequency in healthy people to date. Our data also reveal a further example of sex differences in cough, which warrant additional investigation.

## Data Availability

The datasets used and/or analysed during the current study are available from the corresponding author on reasonable request.

## Declarations

### Clinical Trial Registration

www.isrctn.com; ISRCTN23684347.

## Abbreviations

BMI: Body mass index
CI: Confidence interval
FDA: Food and drug administration
FVC: Forced vital capacity
FEV1: Forced expiratory volume in 1 second
IQR: Interquartile range
REC: Research ethics committee
SD: Standard deviation
